# Commentary: Body Image Distortion and Exposure to Extreme Body Types: Contingent Adaptation and Cross Adaptation for Self and Other

**DOI:** 10.3389/fnhum.2016.00526

**Published:** 2016-10-21

**Authors:** Maria Antonietta Luongo, Mariella Pazzaglia

**Affiliations:** ^1^Department of Psychology, University of Rome “La Sapienza”Rome, Italy; ^2^Clinical Neuroscience Lab, IRCCS Santa Lucia FoundationRome, Italy

**Keywords:** body distortion, multisensory integration, illusions, self-other, body representation

Recent research has informed rich descriptions of the role of visual experience, direct sensory-motor signals, and fundamentally stable hard-wired body representations in forming corporeal experiences and their subsequent effects on behavioral processes and specific cognitive mechanisms (Pazzaglia and Zantedeschi, 2016). The existence of these three mechanisms of body representation (visual, sensory-motor, and temporal constancy of body image) suggests that the reconstruction of one's bodily representation, in fact, results from the integration of multisensory neural inputs (Tsakiris, 2010). In a recent stimulating and timely article, Brooks and colleagues raised some important questions about the role of visual mechanisms in the human ability to process one's own and others' body metrics and distortions of these representations (Brooks et al., 2016). The authors argued that the duration and frequency of visual exposure predict body image adjustments, providing the size estimate of the body has no strong correspondence to one's own body morphology. The study of Brooks and colleagues offers an excellent opportunity to address what differentiates vision from the other senses in term of their roles in body reconstruction and distortions of the self and others.

Perception and feeling that a body is our own is the result of integrate inputs from primarily, interoception but also from vision, somatosensation, and pain that are tightly connected to the motor system. In contrast, the perception of another's body appears to be guided by vision alone. Consequently, visual mapping is used to recognize another person, while multisensory mapping is likely to be the norm in building an image of one's own body (Tsakiris, 2016). Within the visual modality, the perception of self provides a unique (egocentric) body viewpoint that is different to the perspective when one looks at another person (allocentric). This difference may allow our brains to accurately distinguish between the self and others. It is, therefore, important to identify whether or not people move from an allocentric to an egocentric perspective when extracting and projecting self-related body information from photographic images of the self or others on to one's own body. As suggested by Brooks et al., this could lead to the perception of self and other body metric characteristics, which are coded by separate fine grained control mechanisms that are processed by partially overlapping neural circuits in the visual cortex. However, it could also suggest a possible transition of the self to another, which is fundamentally affected by a mechanism of visually induced self-referral (Hodzic et al., 2009a,b).

Another intriguing issue in the context of perception raised by Brooks and colleagues is the visual distortion of the body. Implicit evidence of the formation of real and perceived body dimensions reveals systematic body distortions in healthy populations, suggesting that people do not maintain an accurate body image (Longo, 2016). Moreover, following anesthesia, healthy individuals continue to experience their body as usual, but with more pronounced distortions (Gandevia and Phegan, 1999). The transient effect of anesthesia increases visual body alterations, suggesting that the distortion is not fully determined, but is instead corrected by immediate internal signals (Gandevia and Phegan, 1999). These results suggest that in some ways, in the absence of sensory-motor signals, individuals may have access to less precise body information, and so are more sensitive to body appearance and visual distortions. It is thus necessary to identify whether these distortions are purely visual. This is particularly intriguing when considering therapeutic strategies to correct bodily distortions in various clinical disorders. Multisensory signals forming unique and more accurate corporeal experiences can be used to minimize body image distortion errors (Lucci and Pazzaglia, 2015).

Finally, it is fundamental to explore how visual signals enhance the subjective experience of morphologic similarity between the self and others. The eyes seem to create an image of one's own body and those of other individuals; therefore, visual stimulation can alter self-other boundaries. The occurrence of cross modal illusions, such as the “enfacement illusion” (Tsakiris, 2008) and the “full body illusion,” (FBI; Ehrsson, 2007; Lenggenhager et al., 2007; Petkova and Ehrsson, 2008), indicate that vision, in conjunction with multisensory inputs, can blur identity-defining representations of the self and others, by inducing a perceived psychological “self-other.” Interestingly, in those who experienced FBI, the attribution of a new body to oneself occurred even when it was slimmer or wider than their actual body size (Preston and Ehrsson, 2014). The first person visual perspective appears critical in perceiving this new body as belonging to oneself (Petkova et al., 2011), although, changes in the subjects' corporeal self-awareness occur via a coherent integration of body-centered multisensory information (Petkova and Ehrsson, 2008; Petkova et al., 2011).

However, when body shapes and sizes are altered by means of cross-modal illusory information that shows the body stretching and shrinking, the brain “believes” the modifications in the visual information, giving them precedence over proprioceptive and tactile cues (Kennett et al., 2001; de Vignemont et al., 2005; Preston and Ehrsson, 2014). Rapid and striking changes can thus be induced experimentally (such as full body illusion), producing a more accurate visual image of the body for preventative and therapeutic interventions with respect to distorted body images.

Additionally, healthy humans seem to show a robust tendency to rely exclusively on sight, rather than on their other senses, particularly when defining metric and spatial characteristics. This effect of visual dominance is consistent with the modality appropriateness hypothesis (Aglioti and Pazzaglia, 2010, 2011; Pazzaglia, 2015). From this perspective, the exclusive supremacy of visual information, which is spatially superior to the other senses, seems not to be specific to body perception; instead, it may be determined by information from the modality that provides the most coherent and reliable information reflecting modality-appropriate rules and a more general perceptual trait. Note, however, that given the absence of specific receptors of body metrics, the configuration of the self and others' bodies remains an inherently visual process. Though somewhat imprecise, visual distortions specific to body size are, however, greatly reduced when visual and somatic cues together signaled the body. This suggests that an appropriate decoding of the multisensory system can estimate distortions while also discounting the effects of altered measure perceptions.

Such a mechanism of biased perception for physical similarity might have evolved to support shared body representations, which is a process clearly fundamental for modulating interpersonal reactivity in sociocognitive processing (Gallese and Sinigaglia, 2011). Consequently, given the plasticity of body awareness, it is clear that multiple body representations coexist in the human brain. These respond dynamically and even in a distorted manner to different modalities, which is relevant in the context of illusory bodily resizing (Moseley et al., 2012), the use of functional prostheses (Galli and Pazzaglia, 2015; Galli et al., 2015; Pazzaglia and Molinari, 2016), injury (Fuentes et al., 2013), and pain (Lotze and Moseley, 2007; Pazzaglia et al., 2016), but also in the context of eating disorders, by determining how body metrics are perceived and how we prototypically want to be shaped (Vocks et al., 2011).

## Author contributions

All authors listed, have made substantial, direct and intellectual contribution to the work, and approved it for publication.

### Conflict of interest statement

The authors declare that the research was conducted in the absence of any commercial or financial relationships that could be construed as a potential conflict of interest.
